# Atmospheric-Pressure Cold Plasma Activating Au/P25 for CO Oxidation: Effect of Working Gas

**DOI:** 10.3390/nano8090742

**Published:** 2018-09-19

**Authors:** Jingsen Zhang, Lanbo Di, Feng Yu, Dongzhi Duan, Xiuling Zhang

**Affiliations:** 1 College of Physical Science and Technology, Dalian University, Dalian 116622, China; zhangjingsen0708@163.com (J.Z.); duandongzhi0529@163.com (D.D.); 2 School of Chemistry and Chemical Engineering, Shihezi University, Shihezi 832003, China; yufeng05@mail.ipc.ac.cn

**Keywords:** Au/P25, CO oxidation, atmospheric-pressure cold plasma, working gas

## Abstract

Commercial TiO_2_ (P25) supported gold (Au/P25) attracts increasing attention. In this work, atmospheric-pressure (AP) cold plasma was employed to activate the Au/P25-As catalyst prepared by a modified impregnation method. The influence of cold plasma working gas (oxygen, argon, hydrogen, and air) on the structure and performance of the obtained Au/P25 catalysts was investigated. X-ray diffraction (XRD), UV-Vis diffuse reflectance spectroscopy (DRS), transmission electron microscopy (TEM), and X-ray spectroscopy (XPS) were adopted to characterize the Au/P25 catalysts. CO oxidation was used as model reaction probe to test the Au/P25 catalyst. XRD results reveal that supporting gold and AP cold plasma activation have little effect on the P25 support. CO oxidation activity over the Au/P25 catalysts follows the order: Au/P25-O_2_P > Au/P25-As > Au/P25-ArP ≈ Au/P25-H_2_P > Au/P25-AirP. Au/P25-AirP presents the poorest CO oxidation catalytic activity among the Au/P25 catalysts, which may be ascribed to the larger size of gold nanoparticles, low concentration of active [O]_s_, as well as the poisoning [NO_x_]_s_. The poor catalytic performance of Au/P25-ArP and Au/P25-H_2_P is ascribed to the lower concentration of [O]_s_ species. 100% CO conversion temperatures for Au/P25-O_2_P is 40 °C, which is 30 °C lower than that over the as-prepared Au/P25-As catalyst. The excellent CO oxidation activity over Au/P25-O_2_P is mainly attributed to the efficient decomposition of gold precursor species, small size of gold nanoparticles, and the high concentration of [O]_s_ species.

## 1. Introduction

Supported gold catalysts have attracted increasing research interest after the pioneering work of Haruta [1,2]. Catalytic oxidation of carbon monoxide (CO) over supported gold catalysts has been widely used in indoor air purification, polymer electrolyte membrane fuel cells, low-temperature CO sensors and gas mark [3,4,5,6]. Catalytic performance of the supported gold catalyst is closely related with the size of gold nanoparticles, and the support and preparation method. It is generally accepted that the optimal size of gold nanoparticles is in the range of 2–4 nm [7], and TiO_2_ is the most popular support due to its nontoxicity, low cost, high chemical stability, as well as the strong metal–support interaction formed in CO oxidation [8]. The most adopted method for synthesizing supported gold catalysts is the deposition–precipitation (DP) method. Generally adopted gold precursor (HAuCl_4_) hydrolyzes in solution to form Au(OH)_x_Cl_4−x_^−^. Selection of the support is confined to the isoelectric point [6] to make the positive charged support to absorb the Au(OH)_x_Cl_4−x_^−^ species at a proper rate. Consequently, a simple modified impregnation method is developed and employed to synthesize supported gold catalysts [9]. Both the deposition–precipitation method and modified impregnation method need a post thermal activation process, which may result in the side effect of irreversible gold particles aggregation [10,11]. Therefore, it is urgently necessary to develop an alternative low temperature technique to activate the gold catalysts.

Cold plasma can be operated close to room temperature with high energy electrons, and has been proven to be highly suitable to synthesize supported metal catalysts [12,13,14]. The fast and low-temperature preparation process, and the Coulomb interaction between the charged species and metal precursors ions in cold plasma are conductive to fabrication of metal nanoparticles of small sizes with high dispersion [12]. In addition, the non-thermal equilibrium property of cold plasma is beneficial to enhanced metal–support interaction, amorphous metal nanoparticles [15,16], as well as metal alloy with specific structure [17,18], and metal nanoparticles with specific crystal facet [14]. Thanks to these properties, cold plasma has been successfully employed to prepare and activate gold catalysts, and it has been efficient to enhance the catalytic performance of the gold catalysts [19,20,21,22,23,24,25]. Deng et al. [26] used AP oxygen cold plasma to activate Au/P25 catalysts, and found the prepared catalysts exhibited enhanced activity for visible-light photocatalytic oxidation of CO. In previous work, we adopted AP hydrogen and oxygen cold plasma to synthesize Au/TiO_2_ catalysts, and obtained high performance gold catalysts [27,28]. The effect of discharge time and discharge voltage on the structure and property of the Au/TiO_2_ catalysts are also investigated and discussed [29]. The results indicate that the small size of gold nanoparticles and the high concentration of active surface oxygen species are the main reasons for the high performance. In spite of this, no system work has been carried out to investigate the influence of the cold plasma working atmosphere on the structure and performance of the supported gold catalysts.

In this work, AP cold plasma is adopted to activate the Au/P25-As catalyst prepared by a simple modified impregnation method. The influence of cold plasma working gas (oxygen, argon, hydrogen, and air) on the structure and performance of the obtained Au/P25catalysts was investigated, and the influence mechanism is discussed.

## 2. Materials and Methods

### 2.1. Catalysts Preparation

Chloroauric acid (HAuCl_4_·4H_2_O, ≥99%) purchased from Tianjin Kemiou Chemical Reagent Co. Ltd. was used as gold precursor. Commercial Degussa P25 TiO_2_ obtained from Germany Degussa Corporation was used as support. Aqueous ammonia solution (NH_3_·H_2_O, 25%) was acquired from Liaoning Xinxing Chemical Reagent Co. Ltd. All the chemicals were used as received without any further purification.

Au/P25 catalysts with 1 wt% theoretical gold loading were synthesized according to a modified impregnation method reported in a previous study [9]. First, 2.9 mL deionized water and 1.1 mL HAuCl_4_·4H_2_O solution (C_Au_ = 0.01912 g mL^−1^) were sequentially added into a 5 mL measuring cylinder under continuous stirring. Then, the mixed solution was transferred into a 50 mL beaker with 2 g P25 TiO_2_. The mixture was stirred for a few minutes until it got light yellow and was subsequently aged at room temperature for 12 h. The colloid was rinsed three times with 30 mL aqueous ammonia solution (pH = 11) and three times with 30 mL deionized water in sequence. The product was collected by centrifuge at 10500 rpm for 5 min after each rinse. Finally, Au/P25 catalyst was obtained by drying the product at room temperature for 24 h in a vacuum oven, and was designated as Au/P25-As. The Au content in Au/P25-As is determined to be ca. 0.90 wt% by an Optima 2000DV ICP-AES (Perkin-Elmer, Boston, MA, USA).

**Figure 1:**
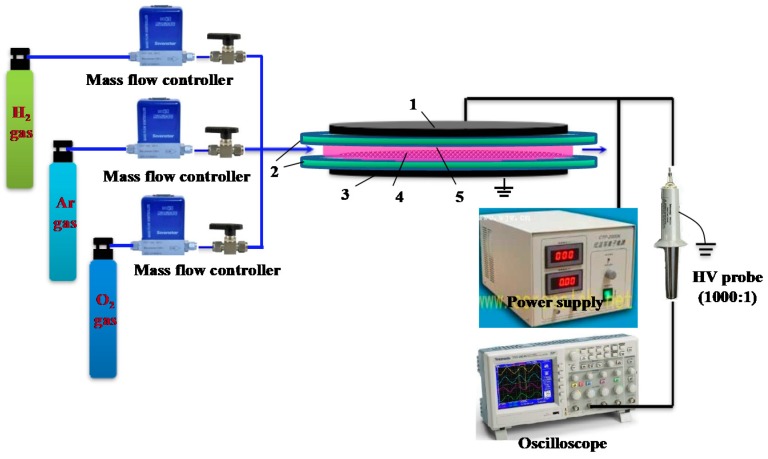
Schematic of the atmospheric-pressure (AP) dielectric barrier discharge (DBD) cold plasma device for activating Au/P25 catalysts. 1-discharge electrode, 2-quartz reactor, 3-ground electrode, 4-sample, 5-cold plasma.

Atmospheric-pressure (AP) dielectric barrier discharge (DBD) cold plasma with various working gases was adopted to activate the Au/P25-As catalyst, and the effect of working gas was investigated. Typically, 0.12 g Au/P25-As was activated by AP cold plasma of oxygen, hydrogen, argon, and air at the discharge voltage of 29 kV for one minute, and the obtained catalysts were denoted as Au/P25-O_2_P, Au/P25-H_2_P, Au/P25-ArP and Au/P25-AirP, respectively. Flow rate of all the working gases were kept at 100 mL·min^−1^. A schematic diagram of the AP DBD cold plasma device is shown in Figure 1. The quartz reactor located between high-voltage electrode and ground electrode consists of an upper quartz plate of 90 mm in diameter and 1 mm in thickness and a quartz circular groove of 4.5 mm in inner depth and 70 mm in inner diameter. Both of the electrodes are made of stainless steel. The discharge frequency and discharge voltage were observed by an oscilloscope (DPO2014, Tektronix, Beaverton, OR, USA) with a 1000:1 high voltage probe (Tektronix, P6015A, Beaverton, OR, USA).

### 2.2. Catalysts Characterization

The crystal structures of the synthesized Au/P25 catalysts were analyzed by X-ray diffraction (XRD) on a DX-2700 X-ray power diffractometer (Dandong Haoyuan, Dandong, China) with Cu Kα radiation (λ = 0.154 nm) at 40 kV and 30 mA. Ultraviolet-Visible (UV-Vis) diffuse reflectance spectroscopy (DRS) was adopted to measure the absorption property of the samples using a U3900 spectrophotometer (Hitachi, Tokyo, Japan). Before testing, the baseline was calibrated using two pieces of BaSO_4_ white plates at the mode of R%. Transmission electron microscopy (TEM) images of the samples were collected on a HT7700 transmission electron microscope (Hitachi, Tokyo, Japan) with an accelerating voltage of 120 kV. The mean sizes of gold nanoparticles and corresponding size distribution were calculated by selecting more than 120 gold nanoparticles from TEM images. Surface chemical analyses of the samples were performed by X-ray photoelectron spectroscopy (XPS) using an ESCALAN250 X-ray photoelectron spectrometer (Thermo VG, Waltham, MA, USA) equipped with a monochromatic Al Kα X-ray source (1486.6 eV photon energy, 150 W). The binding energy of each element in Au/P25 catalysts was calibrated by comparing the standard XPS peak of C1s at 284.6 eV.

### 2.3. Catalytic Activity Evaluation

Catalytic activity of the Au/P25 catalyst was evaluated by CO oxidation in a temperature programmed quartz tube controlled by an electric furnace in the range 30–150 °C. 50 mg Au/P25 catalyst (40–60 mesh) was filled in the middle of a quartz tube with an inner diameter of 4 mm. The catalyst was purged with argon for 15 min prior to reaction. During CO oxidation reaction, the synthetic gas containing 1 vol.% CO, 20 vol.% O_2_ and balance N_2_ was fed into the quartz tube at a flow rate of 20 mL·min^−1^. CO concentration was dynamically monitored by a S710 CO_x_ analyzer (SICK-MAIHAK, Waldkirch, Germany). CO conversion (*X*_CO_) is defined using the following equation:

(1) $$X_{\mathrm{CO}}=\frac{C_{\mathrm{CO}}^{\mathrm{in}}-C_{\mathrm{CO}}^{\mathrm{out}}}{C_{\mathrm{CO}}^{\mathrm{in}}}\times 100\%$$

where $C_{\mathrm{CO}}^{\mathrm{in}}$ and $C_{\mathrm{CO}}^{\mathrm{out}}$ represent the volume concentrations of CO before and after reaction at a certain temperature, respectively.

## 3. Results and Discussion

**Figure 2:**
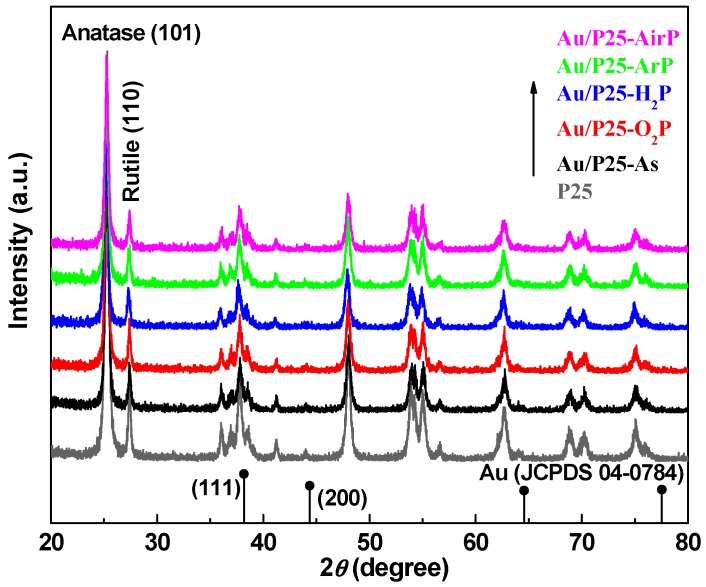
X-ray diffraction (XRD) patterns of the Au/P25 catalysts as prepared and activated by AP cold plasma using various working gases, as well as P25 support.

**Figure 3:**
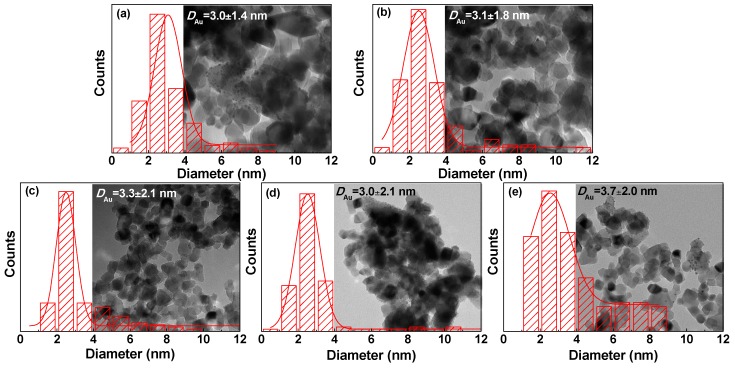
Typical transmission electron microscopy (TEM) images of (**a**) Au/P25-As; (**b**) Au/P25-O_2_P; (**c**) Au/P25-H_2_P; (**d**) Au/P25-ArP; (**e**) Au/P25-AirP, and the corresponding size distribution histograms of gold nanoparticles.

Figure 2 presents the XRD patterns of Au/P25 catalysts as prepared and activated by AP cold plasma with different working gases, as well as P25 support. All the diffraction peaks in the samples can be well indexed as anatase TiO_2_ (JCPDS no.21-1272) and rutile TiO_2_ (JCPDS no. 21-1276). The diffraction peaks at 39.15° and 44.05° can be detected for all the samples including the pure P25 TiO_2_ support, which corresponds to the anatase TiO_2_ (200) and (210) planes (JCPDS no.21-1272). They are very close to the diffraction peaks of Au (111) and (220) planes in the positions of 38.18° and 44.39° (JCPDS no. 04-0784), respectively. Compared to the pure P25 TiO_2_ support, the intensity of these diffraction peaks are decreased for the Au/P25 samples ascribing to the supporting of gold species. Therefore, these peaks should be attributed to anatase TiO_2_ rather than metallic gold. In addition, the nominal loading amount of gold is 1 wt%. The gold species are not detected in the XRD patterns which also indicates that small size of gold species with high dispersion are synthesized, which is consistent with the TEM analysis (*D*_Au_ = 3–4 nm) (Figure 3). Because cold plasma is generated by high-voltage discharge, many researchers are afraid that it may change or destroy the treated materials. Therefore, the influence of AP cold plasma on the structure of P25 TiO_2_ support is discussed based on the XRD data. Positions of the strongest characteristic diffraction peaks of the samples, anatase (101) and rutile (110), were summarized in Table 1. There is no obvious difference among them. To further investigate the influence of supporting gold and AP cold plasma activation on the structure of the P25, the weight fraction and average crystallite size of anatase TiO_2_ and rutile TiO_2_ were also determined, as summarized in Table 1. The weight fraction of rutile TiO_2_ (*W*_rutile_) was obtained according to the following formula [30]:

(2) $$W_{\mathrm{rutile}}=\frac{I_{\mathrm{rutile}}}{0.884I_{\mathrm{anatase}}+I_{\mathrm{rutile}}}$$

where *I*_anatase_ and *I*_rutile_ represent the diffraction intensity of anatase (101) and rutile (110), respectively. The average weight fraction of rutile is 18.2% according to the data listed in Table 1. The crystallite size of anatase TiO_2_ (*D*_anatase_) and rutile TiO_2_ (*D*_anatase_) were obtained according to the Scherrer equation using the characteristic data of anatase (101) and rutile (110). The average crystallite sizes of anatase and rutile TiO_2_ are 21.3 nm and 29.2 nm, respectively. These results indicate that supporting gold and AP cold plasma activation have little effect on the structure of the P25 support.

**Figure 4:**
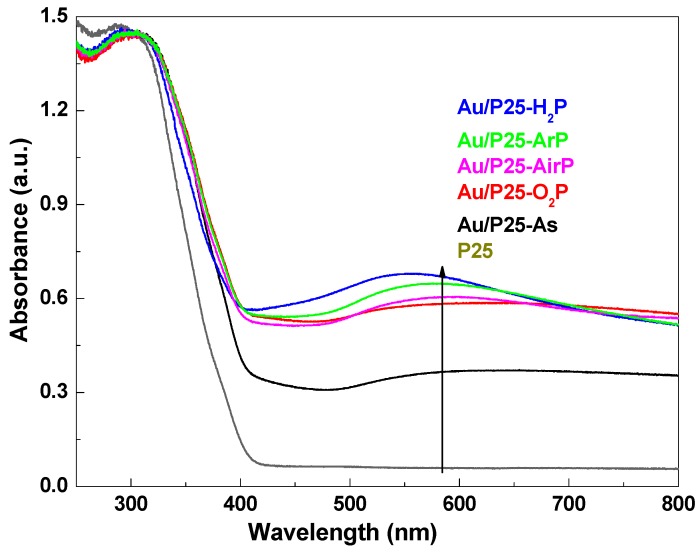
UV-Vis diffuse reflectance spectroscopy (DRS) spectra of the Au/P25 catalysts as prepared and activated by AP cold plasma using various working gases, as well as P25 support.

**Figure 5:**
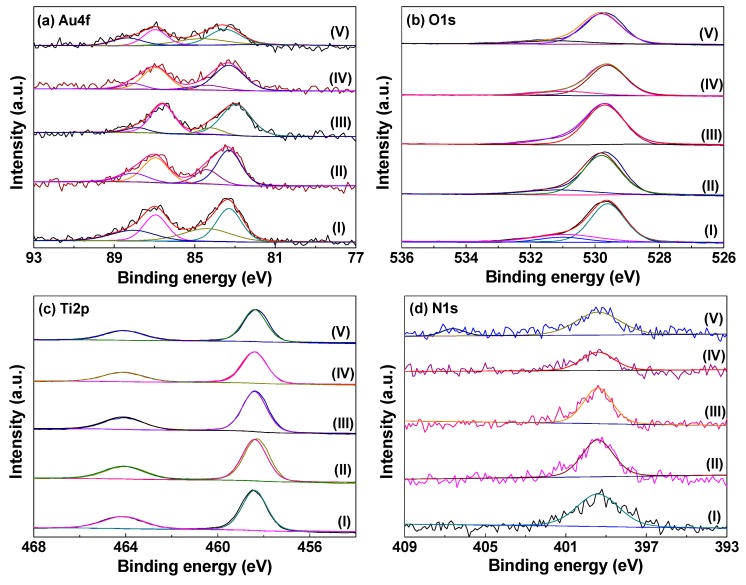
X-ray spectroscopy (XPS) spectra of (**a**) Au4f; (**b**) O1s; (**c**) Ti2p; and (**d**) N1s in (I) Au/P25-As; (II) Au/P25-O_2_P; (III) Au/P25-H_2_P; (IV) Au/P25-ArP; and (V) Au/P25-AirP.

To investigate the optical properties of the samples, UV-Vis DRS spectra of the Au/P25 catalysts as prepared and activated by AP cold plasma with different working gases, as well as P25 support were measured, as shown in Figure 4. For all the samples, the absorption bands at shorter than 400 nm were ascribed to the P25 support [31], while the absorption bands in the visible region were attributed to gold species [32,33]. It is well known that metallic gold nanoparticles irradiated by visible light can lead to Localized Surface Plasmon Resonance (LSPR). A weak LSPR absorption peak for the as-prepared Au/P25-As is also observed, which is consistent with the XPS results (Table 2). The formation of the metallic gold species can be ascribed to the dissociation of the gold precursors due to their photosensitive property. Obviously, compared to Au/P25-As, the absorption for the Au/P25 catalysts activated by AP cold plasma was dramatically enhanced in visible region, and LSPR absorption bands at ca 560 nm were observed due to the high content of metallic gold (Table 2). It confirms that cationic gold species can be reduced into their metallic state by AP cold plasma using various working gases. However, different LSPR absorption signals for the Au/P25 samples are observed, and the intensity of the LSPR peak follows the order: Au/P25-H_2_P > Au/P25-ArP > Au/P25-AirP > Au/P25-O_2_P, which is consistent with the proportion of metallic gold (Table 2) according to the data taken from the result of XPS (Figure 5). The active ground and excited hydrogen atoms generated in cold plasma can not only reduce metal ions with positive standard potential, but also some with negative values [12]. Therefore, the Au/P25-H_2_P demonstrates the most intense LSPR signal. The weakest LSPR absorption peak was observed for Au/P25-O_2_P, which may be ascribed to the strong quenching effect of electronegative oxygen gas on the energetic electrons. It can be verified by the intensity order of the LSPR absorption peak: Au/P25-ArP > Au/P25-AirP > Au/P25-O_2_P.

TEM measurements were carried out to acquire average size and size distribution of gold nanoparticles, which are crucial during the process of gold catalysis [10,34]. Typical TEM images of Au/P25-As, Au/P25-O_2_P, Au/P25-H_2_P, Au/P25-ArP, Au/P25-AirP, and the corresponding size distribution histograms of gold nanoparticles are illustrated in Figure 3. Gold nanoparticles are finely dispersed on the P25 support in the Au/P25 samples. It has to be specified that the gold species are the mixture of oxidized and metallic gold species according to the XPS results (Table 2). Either metallic gold or oxidized gold can be distinguished from the P25 TiO_2_ support in the TEM images due to their contrast ratio. The average gold diameters for Au/P25-As, Au/P25-O_2_P, Au/P25-H_2_P, Au/P25-ArP, and Au/P25-AirP are 3.0 ± 1.4, 3.1 ± 1.8, 3.3 ± 2.1, 3.0 ± 2.1, and 3.7 ± 2.0 nm, respectively (as summarized in Table 2). It was obvious that there is little change in average diameter of the gold nanoparticles for Au/P25-O_2_P, Au/P25-H_2_P, Au/P25-ArP after AP cold plasma treatment. Meantime, the size distribution of gold nanoparticles became a little broader than that of Au/P25-As. These indicate that AP cold plasma did not significantly alter the size and size distribution of gold nanoparticles. However, larger particle size of gold nanoparticles is obtained for Au/P25-AirP after cold plasma treatment. Taking the weak influence of oxygen cold plasma on the size of gold nanoparticles into consideration, larger sizes of gold nanoparticles in Au/P25-AirP may result from the poisoning species [NO_y_]_s_ during nitrogen and oxygen discharge in air cold plasma [24]. This can be confirmed by the XPS spectrum of N1s for Au/P25-AirP (Figure 5d).

The chemical state of gold species, surface oxygen, and other species play important roles in the activity for supported gold catalysts [23,35]. To further investigate the influence of AP cold plasma activation, XPS spectra of Au/P25-As, Au/P25-O_2_P, Au/P25-H_2_P, Au/P25-ArP, and Au/P25-AirP are recorded, as shown in Figure 5. No Cl ions can be detected from the XPS spectra of Cl1s in the Au/P25 samples (not shown here) due to the rinsing of the aqueous ammonia solution and deionized water. In Figure 5a, the XPS spectra of Au4f in these samples can be fitted with two peaks corresponding to metallic Au^0^ and Au^+^ [26], revealing that AP cold plasma can reduce the gold precursor species into metallic state gold. The proportion of metallic Au^0^ and the binding energies of Au4f_7/2_ for these samples are summarized in Table 2. The proportion of the metallic Au^0^ in the samples follows the order: Au/P25-H_2_P > Au/P25-ArP > Au/P25-AirP > Au/P25-O_2_P, which is in line with the intensity sequence of the LSPR peak in UV-Vis DRS spectra (Figure 4). Interestingly, 0.4 eV redshift in the binding energy of Au4f_7/2_ for Au/P25-H_2_P is observed, which can be explained by the following reasons. One the one hand, AP hydrogen cold plasma for synthesizing supported metal catalysts generally may lead to redshift of the binding energy due to the enhanced strong metal–support interaction [18,36]. On the other hand, Au/P25-H_2_P has the highest proportion of metallic Au^0^, which may also enhance the redshift of the binding energy [36].

Surface oxygen species are beneficial to the formation of active intermediates during CO oxidation over supported gold catalysts [6,37]. In Figure 5b, O1s spectra for the Au/P25 samples are illustrated, which can be deconvoluted into two peaks at 529.6 and 530.9 eV, ascribed to crystal lattice oxygen [O]_l_ and surface oxygen species [O]_s_, respectively [38]. Based on individual peak area, [O]_s_ concentration in the oxygen species were calculated and listed in Table 2. The order of [O]_s_ concentration for all the Au/P25 catalysts was Au/P25-As > Au/P25-O_2_P > Au/P25-ArP > Au/P25-AirP > Au/P25-H_2_P, indicating that cold plasma activation can lead to the decline of [O]_s_ concentration and working gas play important roles in [O]_s_ concentration. The significant decrease in [O]_s_ concentration for Au/P25-H_2_P may result from the consumption of [O]_s_ by the hydrogen species [27]. Combined with the results of the proportions of metallic Au^0^ and [O]_s_ (Table 2), it can be concluded that AP cold plasma can not only decompose gold precursor into metallic Au^0^ but also form active [O]_s_ on the P25 surface.

Figure 5c presents the Ti2p XPS spectra of the Au/P25 samples. The peaks at 458.4 and 464.1 eV are attributed to Ti^4+^ of P25 support for the Au/P25 catalysts, confirming that the chemical environment of support didn’t vary after cold plasma activation [23,39]. In Figure 5d, N1s spectra for the Au/P25 catalysts are also investigated. The peaks at 399.4 eV for all the Au/P25 catalysts were ascribed to chemisorbed γ-N_2_ [40]. Interestingly, a new and weak peak at 406.6 eV appeared for Au/P25-AirP, which can be attributed to [NO_x_]_s_ due to the air plasma treatment [24,25]. The atomic ratios of Au/Ti for the Au/P25 samples are determined according to the XPS results and summarized in Table 2. The Au/Ti atomic ratio for Au/P25-As is 0.023. However, they are decreased after AP cold plasma activation due to the dissociation of the gold precursor species. In spite of this, there is no obvious difference for the Au/P25 samples activated by AP cold plasma using different working gases.

**Figure 6:**
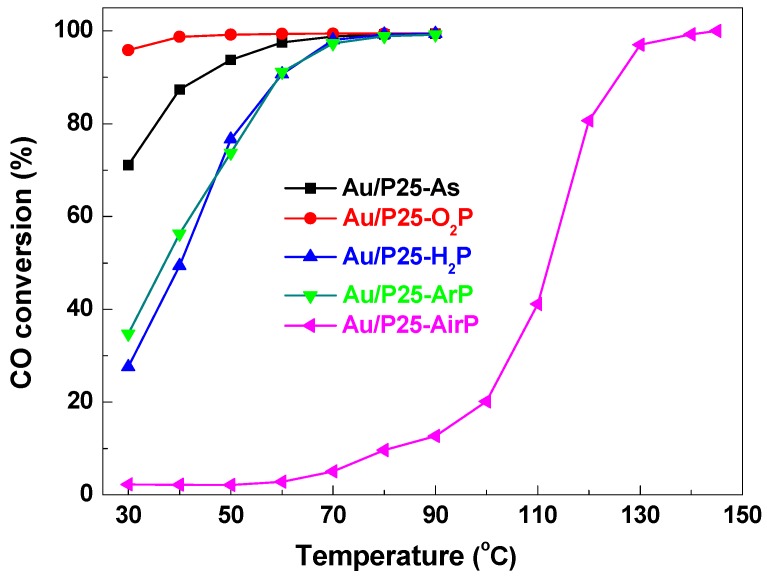
CO conversion over the Au/P25 catalysts activated by AP cold plasma using various working gases, as well as the as-prepared Au/P25-As.

Figure 6 presents CO conversion versus reaction temperature over the Au/P25 catalysts prepared by AP cold plasma activation using various working gases, as well as the as-prepared Au/P25-As. All of the Au/P25 catalysts exhibit high CO oxidation activity. CO oxidation activity over the Au/P25 catalysts follows the order: Au/P25-O_2_P > Au/P25-As > Au/P25-ArP ≈ Au/P25-H_2_P > Au/P25-AirP. Catalytic performance of the Au/P25 catalysts is closely related with the working atmosphere of AP cold plasma. Au/P25-ArP, Au/P25-H_2_P, and Au/P25-AirP obtained by AP argon, hydrogen, and air cold plasma activation show poorer CO oxidation activity than the as-prepared Au/P25-As catalyst, and Au/P25-AirP presents the poorest catalytic activity. Interestingly, AP oxygen cold plasma activation can significantly enhance the catalytic performance of the Au/P25 catalyst. 100% CO conversion temperatures for Au/P25-O_2_P is 40 °C, which is 30 °C lower than that over the as-prepared Au/P25-As catalyst.

AP cold plasma activation with oxygen, argon, and hydrogen as working gas has no significance influence on the size of gold nanoparticles (*D*_Au_ = 3.0–3.3 nm). AP cold plasma activation of the Au/P25-As catalysts may not only lead to the decomposition of the gold precursor species and formation of active [O]_s_ species, but also can result in the removal of active [O]_s_ species after longer AP cold plasma treatment time [29]. The decomposed gold species containing oxidized and metallic gold species can be transformed into active metallic gold species [26]. As a consequence, [O]_s_ species play important roles in CO oxidation. The highest [O]_s_ is observed for Au/P25-As (Table 2). Therefore, it seems that it should exhibit the highest CO oxidation activity. In previous work [26], it has been proved that some [O]_s_ in the as-prepared Au/P25-As sample will be rapidly consumed during the reaction, and lower CO oxidation activity will be obtained. In addition, the high Au/Ti atomic ratio in Au/P25-As is also beneficial to the high CO oxidation activity. For Au/P25-O_2_P prepared by AP oxygen cold plasma activation, the gold precursor species can be well decomposed, and high concentration of [O]_s_ species are obtained. Therefore, Au/P25-O_2_P presents the highest catalytic activity among the Au/P25 catalysts. While, Au/P25-ArP and Au/P25-H_2_P with less concentration of [O]_s_ species show poorer CO oxidation performance. Au/P25-AirP presents the poorest CO oxidation catalytic activity among the Au/P25 catalysts, which may be ascribed to the larger size of gold nanoparticles (*D*_Au_ = 3.7 nm), low concentration of active [O]_s_, as well as the poisoning [NO_x_]_s_ [24,25].

## 4. Conclusions

AP cold plasma was adopted to activate the Au/P25-As catalyst prepared by a modified impregnation method, and the influence of working gas on the structure and performance of the obtained Au/P25catalysts was investigated. XRD analyses confirm that supporting gold and AP cold plasma activation have little effect on the P25 support. All of the Au/P25 catalysts exhibit high CO catalytic oxidation activity. Catalytic performance of the Au/P25 catalysts is closely related with the working atmosphere of AP cold plasma. CO oxidation activity over the Au/P25 catalysts follows the order: Au/P25-O_2_P > Au/P25-As > Au/P25-ArP ≈ Au/P25-H_2_P > Au/P25-AirP. The poor catalytic performance of Au/P25-ArP and Au/P25-H_2_P is ascribed to the lower concentration of [O]_s_ species. Au/P25-AirP presents the poorest CO oxidation catalytic activity among the Au/P25 catalysts, which may be ascribed to the larger size of gold nanoparticles, low concentration of active [O]_s_, as well as the poisoning [NO_x_]_s_. 100% CO conversion temperatures for Au/P25-O_2_P is 40 °C, which is 30 °C lower than that over the as-prepared Au/P25-As catalyst. The excellent CO oxidation activity over Au/P25-O_2_P is mainly attributed to the efficient decomposition of gold precursor species, small size of gold nanoparticles, and the high concentration of [O]_s_ species. AP oxygen cold plasma activation is found to be an efficient method for synthesizing high performance Au/P25 catalysts.

## Figures and Tables

**Table 1:** Structure properties of the Au/P25 catalysts as prepared and activated by AP cold plasma, as well as P25 support.

Samples	2*θ* (degree)		*W*_rutile_ (%)	*D*_anatase_ (nm)	*D*_rutile_ (nm)
	Anatase (101)	Rutile (110)			
P25	25.3	27.5	17.9	21.1	31.0
Au/P25-As	25.2	27.4	17.3	22.3	28.4
Au/P25-O_2_P	25.3	27.4	19.1	21.0	27.9
Au/P25-H_2_P	25.2	27.4	20.1	20.1	29.1
Au/P25-ArP	25.3	27.4	17.2	21.5	28.0
Au/P25-AirP	25.2	27.4	17.4	21.6	31.0

**Table 2:** Gold nanoparticles diameter and XPS data of the Au/P25 catalysts.

Samples	*D*_Au_^a^	Binding Energy (eV)		Proportion (at%)		Au/Ti Atomic Ratios
	(nm)	Au^0^4f_7/2_	Au^+^4f_7/2_	Au^0^/(Au^0^+Au^+^)	[O]_s_/([O]_s_+[O]_l_)	
Au/P25-As	3.0 ± 1.4	83.3	84.4	56.9	27.4	0.023
Au/P25-O_2_P	3.1 ± 1.8	83.3	84.4	71.4	18.9	0.019
Au/P25-H_2_P	3.3 ± 2.1	82.9	84.1	86.2	15.4	0.018
Au/P25-ArP	3.0 ± 2.1	83.3	84.4	81.9	17.3	0.020
Au/P25-AirP	3.7 ± 2.0	83.3	84.5	78.3	16.5	0.018

^a^ The average size of gold nanoparticles was obtained according to the TEM results.
